# Interpretable and Performant Multimodal Nasopharyngeal Carcinoma GTV Segmentation with Clinical Priors Guided 3D-Gaussian-Prompted Diffusion Model (3DGS-PDM)

**DOI:** 10.3390/cancers17223660

**Published:** 2025-11-14

**Authors:** Jiarui Zhu, Zongrui Ma, Ge Ren, Jing Cai

**Affiliations:** Department of Health Technology and Informatics, The Hong Kong Polytechnic University, Hong Kong SAR, China; jiarui.zhu@connect.polyu.hk (J.Z.); zongrui7.ma@connect.polyu.hk (Z.M.)

**Keywords:** nasopharyngeal carcinoma, multimodal segmentation, 3D Gaussian representation, denoising diffusion probabilistic model

## Abstract

**Background:** Gross tumor volume (GTV) segmentation of Nasopharyngeal Carcinoma (NPC) crucially determines the precision of image-guided radiation therapy (IGRT) for NPC. Compared to other cancers, the clinical delineation of NPC is especially challenging due to its capricious infiltration of the adjacent rich tissues and bones, and it routinely requires multimodal information from CT and MRI series to identify its ambiguous tumor boundary. However, the conventional deep learning-based multimodal segmentation method suffers from limited prediction accuracy and frequently performs as well as or worse than single-modality segmentation models. The limited multimodal prediction performance indicates defective information extraction and integration from the input channels. This study aims to develop a 3D Gaussian-prompted Diffusion Model (3DG-PDM) for more clinically targeted information extraction and effective multimodal information integration, thereby facilitating more accurate and clinically interpretable GTV segmentation for NPC. **Methods:** We propose a 3D-Gaussian-Prompted Diffusion Model (3DGS-PDM) that operates NPC tumor contouring in multimodal clinical priors through a guided stepwise process. The proposed model contains two modules: a Gaussian Initialization Module that utilizes a 3D-Gaussian-Splatting technique to distill 3D-Gaussian representations based on clinical priors from CT, MRI-t2 and MRI-t1-contract-enhanced-fat-suppression (MRI-t1-cefs), respectively, and a Diffusion Segmentation Module that generates tumor segmentation step-by-step from the fused 3D-Gaussians prompts. We retrospectively collected data on 600 NPC patients from four hospitals through paired CT, MRI series and clinical GTV annotations, and divided that dataset into 480 training volumes and 120 testing volumes. **Results:** Our proposed method can achieve a mean dice similarity cofficient (DSC) of 84.29 ± 7.33, a mean average symmetric surface distance (ASSD) of 1.31 ± 0.63, and a 95th percentile of Hausdorff (*HD*_95_) of 4.76 ± 1.98 on primary NPC tumor (GTVp) segmentation, and a DSC of 79.25 ± 10.01, an ASSD of 1.19 ± 0.72 and an *HD*_95_ of 4.76 ± 1.71 on metastasis NPC tumor (GTVnd) segmentation. Comparative experiments further demonstrate that our method can significantly improve the multimodal segmentation performance on NPC tumors, with superior advantages over five other state-of-the-art comparative methods. Visual evaluation on the segmentation prediction process and a three-step ablation study on input channels further demonstrate the interpretability of our proposed method. **Conclusions:** This study proposes a performant and interpretable multimodal segmentation method for GTV of NPC, contributing greatly to precision improvement for NPC therapy treatment.

## Simple Summary

This is the first study to utilize 3D Gaussian representations and a Gaussian-prompt diffusion model for performant and interpretable multimodal medical imaging segmentation. Our proposed 3D Gaussian-prompted diffusion model addresses two long-standing challenges in this area: (1) accuracy limitation caused by heavy information redundancy and (2) intepretability defectiveness caused by unreliable information extraction and integration from multimodal inputs. Exclusive experiments have demonstrated that our proposed method can not only evidently boost the multimodal segmentation performance of gross tumor volume for nasopharyngeal carcinoma but also undertake segmentation in an interpretable step-wise diffusion process with traceable contribution from prior guidance on multimodal imaging inputs.

## 1. Introduction

Nasopharyngeal Carcinoma is a malignant tumor arising in the roof and lateral walls of the nasopharyngeal cavity [1]. The majority of NPC tumors are cured with radiation therapy, and image-guided radiation therapy (IGRT) is the standard technique for NPC [2]. During IGRT treatment planning, the delineation accuracy of NPC gross tumor volume (GTV) is crucial due to the proximity between the NPC tumor and critical brain tissues. However, GTV contouring for NPC is particularly error-prone because the NPC can infiltrate diverse areas of the adjacent bones and rich tissues within the head and neck region, and the extent of this involvement is often reflected by subtle changes and exhibits varied contrasts in CT, MRI-t1, MRI-t2 and MRI-t1-contrast-enhancement (MRI-t1-ce) imaging modalities. As a result, NPC tumors are always compounded with the surrounding anatomy, and the tumor boundaries become visually ambiguous and difficult to recognize compared to tumors in other regions.

In clinical scenarios, to delineate accurate contouring of the NPC tumor region, radiologists need to fuse multimodal images (CT, MRI-t1, MRI-t2 and MRI-t1-ce) into the same coordinates, identify anatomical structures by comparing different modalities and adjacent slices, and determine the tumor-contouring workflow based on empirical evidence such as bone infiltration in MRI-t1, lymph node enlargement in MRI-t2 and meninges thickening in MRI-t1-cefs [3]. The clinical delineation process is extremely labor-intensive and highly reliable to the radiologists’ expertise.

To reduce the workload, fast and accurate deep-learning-based NPC GTV segmentation methods have gained overwhelming popularity in recent years. Conventional deep learning-based models follow an annotation-targeting paradigm by formulating deterministic mapping from the source image to the predicted segmentation masks. Segmentation models based on a single modality source image, such as CT imaging or MRI-t1-ce, have achieved moderate segmentation accuracy [4,5]. These methods seek network complexity increments and the integration of clinical priors to improve the segmentation accuracy. However, due to the diverse information exhibited by the NPC on CT and different MRI series, the incomplete anatomical information from a single modality have drawn serious clinical concerns and potentially hampers the segmentation performance.

Great efforts have been devoted to developing a multimodal segmentation model for further segmentation accuracy increments, such as multi-scale sensitive U-Net [6], intuition-inspired hypergraph modeling [7], automatic weighted dilated convolutional network [8], enhancement learning [9], mask-guided self-knowledge distillation [10], uncertainty and relation-based semi-supervised learning [11] and uncertainly-based end-to-end learning [12]. However, these attempts have achieved limited improvement or even performance degradation. Information redundancy may be the fundamental reason behind the performance degradation, as although supplementary tumor-related information is integrated from multimodal inputs, a larger ratio of irrelevant information is introduced at the same time [13]. The high ratio of irrelevant information greatly increases training difficulty [14], and the models fail to efficiently extract tumor-focused information from multimodal inputs while comprehensively integrating pertinent information for more accurate tumor contouring. Most importantly, due to the simplicity of the high-dimensional convolutional feature space and end-to-end predicting process, there remains an urgent demand for methods which can effectively undertake clinically oriented information extraction and reasonable contribution distributions from multimodal inputs.

To address the above limitations, in this study, we propose a 3D Gaussian-prompted Diffusion Model (3DG-PDM) for accurate and reasonable multimodal NPC segmentation. The 3DG-PDM contains two modules: a Gaussian Initialization Module that utilizes a 3D-Gaussian-Splatting technique to distil 3D-Gaussian representations based on clinical priors for clinically oriented information extraction from CT, MRI-t1-contract-enhanced(MRI-t1-ce) and MRI-t2, respectively, and a diffusion segmentation module that generates tumor segmentation step by step, guided by step-wise 3D-Gaussian, for an interpretable segmentation process. Extensive experiments are conducted on 600 NPC patients from four hospitals to evaluate the segmentation performance of our model. The dice similarity coefficient (DSC), mean average symmetric surface distance (ASSD) and 95th percentile of Hausdorff (*HD*_95_) indexes are employed for quantitative evaluation compared to other state-of-the-art multimodal segmentation models. A visual evaluation and ablation study are also performed to demonstrate the performance and interpretability of our method.

## 2. Materials and Methods

### 2.1. Method Overview

**Figure 1:**
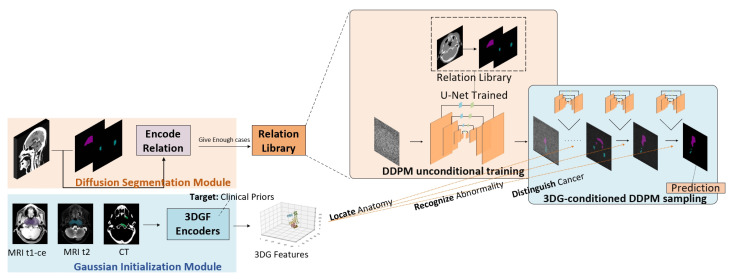
The overall workflow of the proposed method.

Figure 1 shows the overall workflow of the proposed 3DG-PDM model. A diffusion segmentation model is pretrained first, to build a relation library encoding segmentation mapping relation between source CT imaging and the paired GTV clinical annotations. Then, a Gaussian initialization module is pretrained on CT, MRI-t1-ce and MRI t2 imaging to extract 3D Gaussian features (3DGF) which contain key information from multimodal inputs, based on clinical focuses on each modality. During the segmentation process, multimodal imaging data (MRI-t1-ce, MRI-t2 and CT) are fed into 3DGF encoders to extract clinically-focused Gaussian features, and then the Gaussian features are used as prompts for guiding the conditional sampling process of the diffusion segmentation module, generating stepwise coarse-to-fine segmentation predictions with precise anatomy location, abnormality recognition and cancer distinguishing ability.

### 2.2. Gaussian Initialization Module

The Gaussian initialization module refines key information from multimodal imaging inputs based on clinical priors guidance, which filters unwanted information and facilitates effective multimodal information extraction.

Inspired by the success of the 3D Gaussian splatting technique in the area of novel 3D scene reconstruction [15], a great deal of research has been developed to leverage the unique advantages of 3D Gaussian feature segmentation [16,17,18]. Compared to conventional high-dimensional feature spaces encoded by convolutional neural network (CNN) layers or transformer tokens, features encoded in 3D Gaussian space have unique advantages: (1) 3D Gaussian features are highly refined from input imaging owing to the pixel-to-Gaussian training process; (2) 3D Gaussian features can be extracted individually on a single imaging modality integrated from multimodal imaging without information loss, through mathematical addition between 3D Gaussian distributions; and (3) 3D Gaussian features are extracted and applied in a clinically interpretable process, because the information containment of 3D Gaussian features is observable in structured low-dimensional Gaussian space. The explicitness of 3D Gaussian features enables effective information extraction from clinical priors.

**Figure 2:**
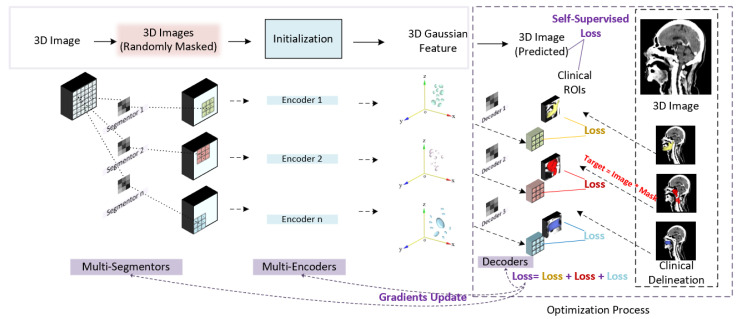
Gaussian initialization module.

Based on the above traits of 3D Gaussian features, we employ a 3D Gaussian features extraction technique for refining clinically focused information from multimodal inputs. To adapt the 3D Gaussian feature extraction process in medical imaging scenarios, we designed a Gaussian initialization module. Figure 2 shows the architecture of the proposed Gaussian initialization module. Regional 3D points are first located from a 3D image input by multiple CNN layers, namely multi-segmentors. An initialization block containing multiple 3D Gaussian encoders (3DGE) is then applied to transfer pixel points into 3D Gaussian feature points. The 3D Gaussian feature points are then decoded into regional anatomy images by multiple CNN layers, namely decoders. Finally, similarity loss is calculated by comparing decoded regional anatomy images and real regional anatomical images acquired based on clinical delineations. By optimizing the similarity loss, the gradients of the network are updated.

Specifically, the initialization block initials 3D Gaussian feature points from pixel points referring to definitions from the original 3D Gaussian splatting techniques [15]. The definition of each 3D Gaussian feature point can be formulated as

(1) $$G\left(x\right)=e^{-\frac{1}{2}{\left(x-\mu \right)}^{T}\sum^{-1}\left(x-\mu \right)}C\left(\theta ,\phi \right)$$

where *x* represents a pixel point in 3D space and G(x) represents a 3D Gaussian point in 3D Gaussian space. μ represents the mean position of the Gaussian feature point in 3D Gaussian feature space. C(θ,ϕ) refers to Spherical Harmonic coefficients, which represent color information.

The 3D Gaussian features are conventionally rendered to 2D images by a “splatting” technique; they are projected to 2D Gaussian features by transforming the covariant matrix $\sum^{\prime }$ according to the following transformation [19]:

(2) $$\sum^{\prime }=JW\sum W^{T}J^{T}$$

where *J* represents the Jacobian of the affine approximation of the projective transformation, and W is the view transformation.

Since medical imaging is defined by pixel intensity rather than RGB colors, we alters the 3D Gaussian feature formulation according to [20] as follows:

(3) $$G\left(x\right)=e^{-\frac{1}{2}{\left(x-\mu \right)}^{T}\sum^{-1}\left(x-\mu \right)}i$$

where *i* refers to pixel intensity value.

Additionally, compared to rendering 3D Gaussian feature points into 2D images, we employ CNN decoders for directly transforming 3D Gaussian feature points into 3D imaging.

### 2.3. Diffusion Segmentation Module

The diffusion segmentation module integrates multimodal information in a Gaussian-prompted step-wise sampling process. The integration of Gaussian features in Gaussian space enables more effective information fusion, and the combination of Gaussian prompts and diffusional sampling processes allows segmentation to operate in an interpretable way. Interpretively, the influence of Gaussian prompts is explicitly exhibited as the altering of the sampling trajectory. This altering further demonstrates that 3D Gaussian features can effectively extract clinical prior information, and multimodal prior information can effectively alters segmentation processes through different aims.

**Figure 3:**
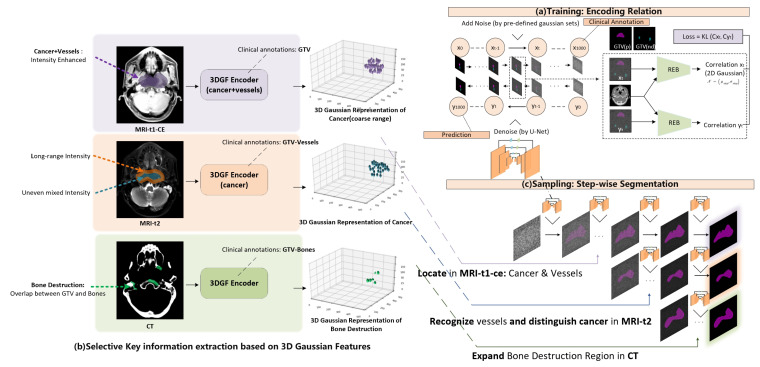
Diffusion segmentation module.

Figure 3 shows the architecture of the proposed diffusion segmentation module. Figure 3a shows the unconditional pre-training process of the image-GTV relation library encoding. A typical U-Net is employed for denoising, and special relation-encoding blocks (REB) are used for generating correlations from CT imaging and GTV mask predictions, with reference to conventional designs of diffusion models for segmentation [21].

Figure 3b shows the key information selection process during 3D Gaussian feature extraction. According to an influential clinical NPC segmentation guideline [22], during the clinical NPC delineation process, doctors have a strong empirical focus on multimodal imaging. For NPC patients, due to mucosal inflammation, the water region is visually dark in MRI t1-ce imaging, resulting in an intensity enhancement of the cancer region and vessels. Furthermore, a combined region containing cancer and vessels can be further recognized from MRI-t1-ce by thresholding. The enhanced region automatically acquired by thresholding can also be used to guide 3D Gaussian feature extraction from MRI-t1-ce imaging to encode a coarser cancer range combining the cancer and vessels. Additionally, while the combined region of vessels and cancer is still intensity-enhanced, the vessels experience long-duration highlighting in MRI-t2 imaging, although the intensity distribution of the cancer region is uneven and mixed. This distribution difference can be leveraged to distinguish cancer from vessels, and can allow fine cancer contouring to be acquired from a coarse cancer range. To guide the 3D Gaussian feature encoder to recognize vessel regions, we set the training target as the difference found between the intensity-enhanced region (acquired by thresholding adjustment) and clinically annotated GTV. In addition, because NPC cancer involves bone destruction and CT exhibits high-contrast bone information, a 3D Gaussian feature encoder on CT imaging was developed for locating the bone-destructed region, targeted by an overlap between GTV annotations and bones (acquired by thresholding adjustment).

After pre-training of the diffusion segmentation module, which encodes the CT-GTV relation, and the pre-training of the Gaussian initialization module, which encodes multimodal clinically-focused priors, a Gaussian-prompted step-wise segmentation is operated during the sampling process of the diffusion model. As can be seen from Figure 3c, as GTV contouring is generated step-wise from the denoising sampling process of the diffusion model, 3D Gaussian features extracted from multimodal inputs are used as prompts to perturb the step-wise segmentation process. During this conditional sampling process, a coarse cancer range is firstly located by Gaussian features from MRI-t1-ce imaging, finer cancer contouring is then recognized by Gaussian features from MRI-t2 imaging, and finally the generated GTV contouring is expanded to include bone destruction regions through Gaussian features from CT imaging. By combining the Gaussian initialization module and the diffusion segmentation module, the proposed 3DG-PDM is able to operate interpretable and performant multimodal NPC GTV segmentation in a step-wise auto-contouring process guided by 3D Gaussian feature-encoded clinical priors.

Owing to its mathematical advantages, a 3D Gaussian distribution can be mathematically reflected along one axis into 2D Gaussian distribution. During the conditional sampling process, we reflected our extracted 3D Gaussian feature points along the z-axis into 2D Gaussian distribution and added them into step-wise Gaussian noise in the conditional sampling process. Compared to conventionally adding the image as a condition, directly adding 2D Gaussian distribution achieves better information preservation. To further enable prompts from multi-modal inputs to guide the sampling process, we added the condition step-wise from the three input modalities to the sampling process, specifically 3DG from MRI-t1-ce in step 1–350, 3DG from MRI-t2 in step 351–700 and 3DG from CT in step 701–1000.

The visibility of the input-feature and feature-prediction corresponding relations during the generating process strongly demonstrate the contribution of each imaging modality and inherently improve the segmentation performance through more refined information leveraging.

### 2.4. Dataset

**Figure 4:**
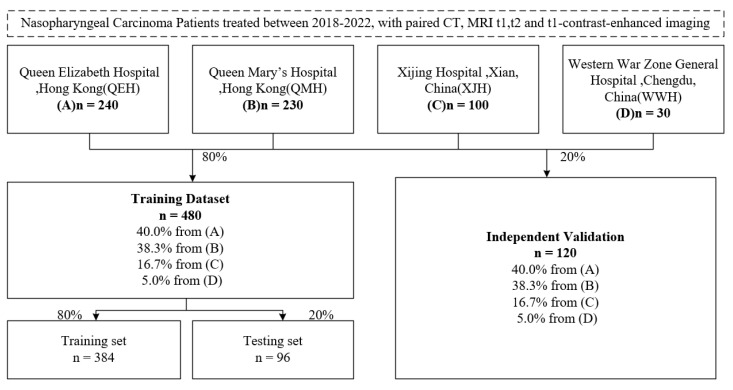
Dataset description flow.

As can be seen from Figure 4, data on 600 NPC patients were retrospectively collected with CT, MRI series (t1, t1-contrast enhanced and t2) and clinical annotations from four hospitals: Queen Mary’s Hospital, Hong Kong (QMH); Queen Elizabeth Hospital, Hong Kong (QEH); Xijing Hospital, Xian, China (XJH); and Western War Zone General Hospital, Chengdu, China (WWH). Then, 480 cases were selected as the training set and 120 cases for independent testing. The training set was further separated randomly into 384 cases for training and 96 cases for validation. For each patient, to align the MRI series and CT imaging into the same spatial coordinate system, 3D registration was operated using the Elastix registration toolbox from the MRI series (moving images) to CT imaging (fixed image) [23]. The voxel units of both CT and MRI series imaging were resampled to a 1:1:1 ratio by spacing along x, y and z axes. The intensity values of all images were scaled to the range of [0, 1], with a scaling factor calculated by the difference between the minimum and maxium intensity value, respectively, from all CT images and from each MRI imaging series. For CT imaging, a Hounsfield Unit (HU) value below −1000 or over 1000 was replaced with −1000 or 1000.

### 2.5. Implementation Details

All experiments were conducted on a 48 GB A6000 GPU. The number of 3D Gaussian feature points were initialized as 50,000 for each input imaging modality, similar to [20], occupying around 20GB GPU space during the Gaussian feature initialization training stage and 1 GB GPU space at the Gaussian feature initialization inference stage. The diffusion model for segmentation was unconditionally trained on 3D volumes of size (256,256,20) along the x, y and z axes, occupying 16GB GPU space for each individual batch.

## 3. Results

### 3.1. Quantitative Evaluation

To demonstrate the superiority of our method, we compared it with five state-of-the-art multimodal segmentation methods: MsU-Net [6], AD-Net [8], Multi-resU-Net [24], nnformer [9] and nnU-Net [25]. The dice similarity coeffient (DSC) [26], average symmetric surface distance (ASSD), and 95th percentile of Hausdorff ($HD_{95}$) indexes were calculated between the predicted GTV segmentation and the real clinical annotations. The quantitative comparison results are listed in Table 1.

The table shows that our method achieved the highest segmentation accuracy among all comparative multimodal segmentation methods. Our method achieved a mean DSC of 84.29 ± 7.33 for GTVp segmentation and a mean DSC of 79.25 ± 10.01 for GTVnd segmentation, and reached an average segmentation accuracy of 81.77 ± 8.67. As observed, for GTVp segmentation, the mean DSC of comparative methods such as AD-Net, Multi-resU-Net, nnformer and nnU-Net commonly meets a bottleneck at 80. Compared to these methods, our method has achieved a significant accuracy improvement to 84, and the dominant accuracy improvements indicate that our method breaks through the performance bottleneck due to more efficient multimodal information extraction and integration. For GTVnd segmentation, our method also achieved prominent accuracy improvement. ASSD and $HD_{95}$ indexes follow the same performing tendency as DSC, which further demonstrates the superiority of our method.

### 3.2. Qualitative Evaluation

**Figure 5:**
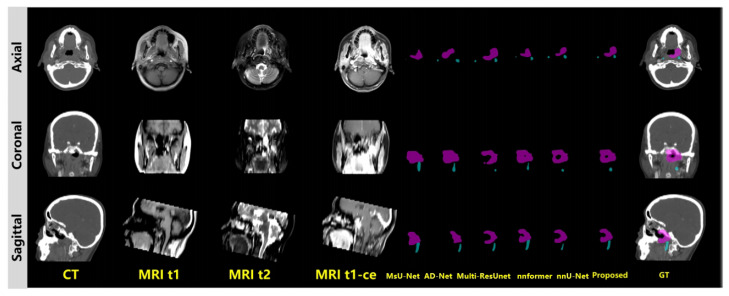
Visual evaluation.GTVp is marked in pink and GTVnd is marked in blue.

For the qualitative evaluation, a representative case was selected for evaluating the visual performance of our model. Figure 5 shows the comparative visual performance evaluation, including GTVp and GTVnd segmentation results. GTVp is marked in pink and GTVnd is marked in blue. The figures show that the GTVp and GTVnd contouring generated by our proposed method achieved the best similarty to the ground truth clinical annotations. It is worth noticing that our method is able to recognize subtle details within the cancer region, and achieves stable segmentation accuracy on both GTVp and GTVnd. By contrast, comparative methods have great difficulty in distinguishing subtle health regions from the cancer range, and most of them fail to maintain stable performance for both GTVp and GTVnd.

### 3.3. Ablation Study

**Figure 6:**
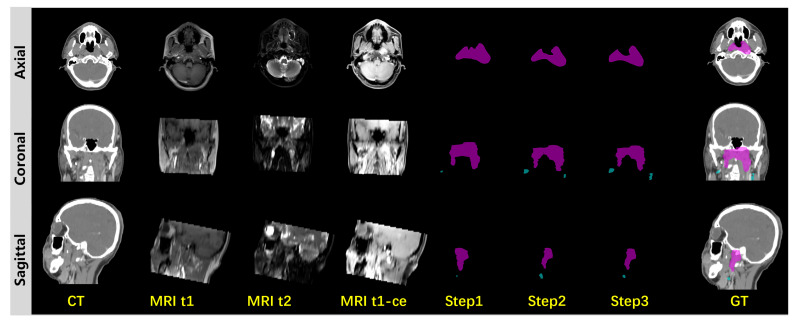
Ablation study.GTVp is marked in pink and GTVnd is marked in blue.

To further demonstrate the effectiveness of multimodal information extraction and integration, we conducted an ablation study. As is shown in Figure 6, in the ablation study, the multimodal input channels are gradually fed to the diffusion model in three steps. Step 1 involves single Gaussian feature prompting from MRI-t1-ce imaging during the diffusion segmentation sampling process. Step 2 uses both Gaussian features from MRI-t1-ce and MRI-t2 imaging to prompt the diffusion sampling process. In step 3, Gaussian features from CT are added for a complete three-step prompt from all MRI-t1-ce, MRI-t2 and CT source imaging data. From the three-step ablation process we can observe improvements in segmentation accuracy, from a coarse cancer range to fine and even finer cancer contouring. We summarized the quantitative experiments on the ablation study in Table 2. The ablation study evidently demonstrates the effectiveness of information extraction and integration from multimodal imaging inputs. We summarized the quantitative results in Table 2, with a comparison between our proposed method and other multimodal segmentation methods by DSC, ASSD and $HD_{95}$.

## 4. Discussion

In this study, we proposed a 3D Gaussian-prompted Diffusion Model for performant and interpretable multimodal NPC segmentation. We designed a 3D Gaussian feature extraction strategy, namely the Gaussian extraction module, for selectively extracting clinically focused information from multimodal imaging. Additionally, we designed a Gaussian-prompted segmentation-oriented denoising diffusion probabilistic model, namely the diffusion segmentation module, to efficiently integrate multimodal information and undertake segmentation in an interpretable step-wise generating process.

Exclusive experiments have demonstrated that, compared to other methods, our proposed method has achieved an accuracy improvement on both GTVp and GTVnd segmentation by DSC, on a multi-institutional testing dataset built on four hospitals. While other methods reached their performance bottleneck at a average DSC of 77, our method has made a significant breakthrough by increasing the average DSC to 81. ASSD and $HD_{95}$ indexes follow the same performing tendency as DSC. As has been mentioned in many works focusing on multimodal generation [13,27,28], information redundancy could be the main cause hampering the model performance. We addressed this limitation by proposing a Gaussian initialization module for a more refined and clinically biased information extraction from the multimodal inputs. The proposed Gaussian initialization module fully leverages the unique advantages of 3D Gaussian representations, including key clinically focused information refinement based on the highly refined trait of 3D Gaussian feature points, efficient multimodal information integration based on flexible mathematical additions in Gaussian space, and interpretable coarse-to-fine segmentation predicting processes based on the explicitness of low-dimensional Gaussian feature and the compatibility between Gaussian prompts and the conditional diffusion model. Additionally, the quantitative analysis summarized in Table 1 showed that, compared to other methods, our proposed method achieved sufficient performance consistency among four different hospitals with the minimum center bias. The high multi-institutional consistency further demonstrated the generalization ability and high clinical utility of the proposed method. Another unique advantage of the proposed method is that information is extracted separately from each input modality channel, which greatly alleviates potential registration errors and inherently increases the generalization ability across different centers.

Not restricted to more refined and clinically reliable information extraction, our proposed Gaussian-prompted diffusion segmentation module also further endows the whole multimodal segmentation process with interpretability. As related works commonly point out that the contribution of multimodal input channels tends to become uneven during the training process [29], currently, deep learning-based methods may not be able to achieve effective information integration from multimodal inputs based on a real clinical focus. This ineffective information integration can greatly limit the model’s performance, and moreover, adjusting the multimodal information contribution can be challenging due to the implicit end-to-end training paradigm. Adding multichannel weighing factors [30] or designing loss functions for specific channels [31] are common solutions for adjusting multichannel channel contributions, yet these methods lack effectiveness and fail to utilize clinical priors for adjusting multimodal input contributions. Combining Gaussian prompts and diffusion models can ensure interpretable and effective multimodal information integration. On the one hand, clinical bias can be applied in the Gaussian feature extraction period, for example, by guiding the Gaussian encoder to extract bone-destruction-related information from CT imaging. The clinically biased information can be further conveyed to the segmentation process by adding the Gaussian feature to the intermediate images during the sampling process of the diffusion model, without information loss due the flexible operational ability of Gaussian distribution. On the other hand, clinically biased information from different imaging modalities can be respectively used for prompts during the sampling process, and the effects from the prompt to the segmentation trajectory altering can be observable. Compared to a conventional end-to-end segmentation model, diffusion model-based segmentation is undertaken in an observable step-wise denoising trajectory [32], and the altering of the trajectory path prompted by Gaussian features from multimodal inputs reasonably demonstrates the multimodal information integration effectiveness and further demonstrates the interpretability of the proposed method. The conducted ablation study evidently reveals this effectiveness and interpretability.

Although our proposed method has achieved considerable accuracy improvement and demonstrated interpretability for multimodal segmentation, it still suffers from slow generating speed due to the diffusion-based model design. Additionally, there is still a wealth of professional knowledge on the clinical NPC GTV delineation process that can be leveraged in the Gaussian feature extract stage for a more clinically focused design. The multi-institutional robustness of the proposed method also requires further inter-observer assessments. In the future, we plan to employ more advanced denoising diffusion probabilistic approaches for accelerating the segmentation generation process, such as denoising diffusion implicit models (DDIM). We also plan to further explore effective clinical knowledge on recognizing NPC GTV in different imaging modalities for more comprehensive Gaussian initialization module design, thereby further boosting the model performance.

## 5. Conclusions

This study introduces a 3D Gaussian-prompted diffusion model for multimodal gross tumor volume (GTV) segmentation of nasopharyngeal carcinoma (NPC) patients. The model is designed to enhance the clinical relevance of feature extraction process and improve the integration efficiency of multimodal imaging information fusion process. The proposed method addresses two long-standing challenges in multimodal segmentation:accuracy limitation due to information redundancy;and poor interpretability caused by unreliable data fusion process. Extensive experiments demonstrate that the proposed model achieves significant improvements in both accuracy and interpretability over state-of-the-art methods. These gains are attributed to its use of 3D Gaussian representations for more clinically targeted feature extraction and a Gaussian-prompted diffusion mechanism for more effective multimodal integration. Overall, this work offers a robust and interpretable tool for multimodal NPC GTV segmentation, which is critical for NPC radiation therapy treatment precision. The proposed interpretable and robust multimodal segmentation paradigm processes significant clinical utility, which may potentially facilitate a more effective image-guided radiation therapy process.

## Figures and Tables

**Table 1:** Comparison between our method and other multimodal segmentation methods.

		DSC (%)						ASSD (mm)						${\mathrm{HD}}_{95}$ (mm)					
Method	Institution	GTVp		GTVnd		Average		GTVp		GTVnd		Average		GTVp		GTVnd		Average	
		Mean	Dev	Mean	Dev	Mean	Dev	Mean	Dev	Mean	Dev	Mean	Dev	Mean	Dev	Mean	Dev	Mean	Dev
	QEH	75.10	7.08	69.21	7.99	72.16	7.54	1.23	0.35	0.96	0.53	1.10	0.44	3.87	2.84	3.09	2.64	3.48	2.74
	QMH	78.27	10.62	70.93	7.74	74.60	9.18	1.20	0.49	0.87	0.44	1.03	0.47	3.46	2.97	2.94	3.22	3.20	3.10
Msu-Net	XJH	75.31	7.79	73.50	7.34	74.41	7.57	1.12	0.61	1.18	0.41	1.15	0.51	3.75	3.26	2.91	2.65	3.33	2.96
	WWH	79.24	2.07	74.20	7.17	76.72	4.62	1.41	0.43	1.19	0.86	1.30	0.65	3.40	3.65	3.94	3.45	3.67	3.55
	**Average**	76.98	6.89	71.96	7.89	74.47	7.39	1.24	0.47	1.05	0.56	1.15	0.52	3.62	3.18	3.22	2.99	3.42	3.09
	QEH	80.88	9.90	72.90	9.67	76.89	9.79	1.27	0.56	1.20	0.30	1.23	0.43	3.74	2.13	3.45	1.62	3.60	1.88
	QMH	81.79	4.24	71.78	9.94	76.79	7.09	1.11	0.65	1.15	0.45	1.13	0.55	4.08	1.96	3.40	1.53	3.74	1.75
AD-Net	XJH	82.03	8.89	74.87	13.02	78.45	10.96	1.01	0.57	0.92	0.28	0.97	0.43	3.82	1.93	3.07	1.66	3.45	1.80
	WWH	76.74	13.81	79.53	16.01	78.13	14.91	1.65	0.66	1.13	1.09	1.39	0.88	4.32	3.02	3.52	2.27	3.92	2.64
	**Average**	80.36	11.66	74.77	12.16	77.57	11.91	1.26	0.61	1.10	0.53	1.18	0.57	3.99	2.26	3.36	1.77	3.68	2.01
	QEH	80.57	5.16	71.42	9.59	76.00	7.38	1.30	0.42	1.01	0.37	1.16	0.40	3.84	2.49	3.27	2.28	3.56	2.38
	QMH	81.74	5.85	73.00	9.70	77.37	7.78	1.37	0.33	1.23	0.47	1.30	0.40	3.72	2.78	3.02	2.26	3.37	2.52
Multi-resU-Net	XJH	84.00	7.72	73.74	8.05	78.87	7.89	1.31	0.39	1.20	0.58	1.25	0.49	3.82	2.67	3.36	2.06	3.59	2.37
	WWH	81.65	19.83	74.72	9.78	78.18	14.81	1.02	0.86	0.92	0.66	0.97	0.76	3.66	2.74	2.87	1.72	3.26	2.23
	**Average**	81.99	9.64	73.22	9.28	77.60	9.46	1.25	0.50	1.09	0.52	1.17	0.51	3.76	2.67	3.13	2.08	3.45	2.38
	QEH	82.43	6.12	75.82	7.56	79.13	6.84	1.33	0.70	1.16	0.41	1.25	0.55	4.66	1.82	4.30	2.00	4.48	1.91
	QMH	82.45	9.09	74.33	11.12	78.39	10.11	1.07	0.41	1.20	0.25	1.14	0.33	4.71	1.70	4.00	2.48	4.36	2.09
nnformer	XJH	74.81	7.58	76.48	11.52	75.65	9.55	1.32	0.48	1.22	0.46	1.27	0.47	4.28	1.56	4.26	2.16	4.27	1.86
	WWH	81.47	12.37	72.93	8.88	77.20	10.62	1.36	0.85	1.02	0.84	1.19	0.85	4.55	1.16	4.16	2.36	4.35	1.76
	**Average**	80.29	8.79	74.89	9.77	77.59	9.28	1.27	0.61	1.15	0.49	1.21	0.55	4.55	1.56	4.18	2.25	4.37	1.91
	QEH	80.50	8.13	75.77	11.43	78.13	9.78	1.05	0.42	1.00	0.61	1.02	0.52	4.24	1.72	3.93	1.40	4.09	1.56
	QMH	83.30	6.17	74.65	10.20	78.97	8.18	1.38	0.39	1.25	0.47	1.32	0.43	4.27	1.56	4.27	1.50	4.27	1.53
nnU-Net	XJH	79.07	4.27	77.94	13.48	78.51	8.88	1.25	0.68	1.09	0.59	1.17	0.64	4.36	1.85	4.34	1.78	4.35	1.82
	WWH	83.61	14.31	72.52	6.33	78.07	10.32	1.44	0.87	1.10	0.93	1.27	0.90	5.29	1.95	4.38	1.56	4.84	1.76
	**Average**	81.62	8.22	75.22	10.36	78.42	9.29	1.28	0.59	1.11	0.65	1.20	0.62	4.54	1.77	4.23	1.56	4.39	1.67
	QEH	82.51	4.89	81.94	9.40	82.23	7.15	1.34	0.52	1.22	0.66	1.28	0.59	4.86	1.81	4.48	1.68	4.67	1.75
	QMH	82.08	5.06	78.05	12.55	80.07	8.81	1.32	0.45	0.94	0.51	1.13	0.48	4.58	1.85	4.31	1.70	4.45	1.78
Proposed	XJH	84.86	4.40	80.70	10.48	82.78	7.44	1.09	0.42	0.98	0.74	1.04	0.58	4.95	1.73	4.49	1.51	4.72	1.62
	WWH	87.71	14.97	76.31	7.97	82.01	11.47	1.49	1.13	1.62	0.97	1.56	1.05	4.65	2.53	5.16	1.95	4.91	2.24
	**Average**	**84.29**	7.33	**79.25**	10.10	**81.77**	8.72	**1.31**	0.63	**1.19**	0.72	**1.25**	0.68	**4.76**	1.98	**4.61**	1.71	**4.69**	1.85

**Table 2:** Quantitative ablation study with comparison between the proposed method and other multimodal segmentation methods.

Method	Institution	Step 1 MRI-t1-ce			Step 2 MRI-t1-ce, MRI-t2			Step 3 MRI-t1-ce, MRI-t2, CT		
		DSC (%)	ASSD (mm)	${\mathrm{HD}}_{95}$ (mm)	DSC (%)	ASSD (mm)	${\mathrm{HD}}_{95}$ (mm)	DSC (%)	ASSD (mm)	${\mathrm{HD}}_{95}$ (mm)
	QEH	71.04	0.94	3.41	71.63	1.02	3.45	72.16	1.10	3.48
	QMH	73.32	0.85	3.18	74.01	0.95	3.19	74.60	1.03	3.20
Msu-Net	XJH	73.26	1.01	3.29	73.87	1.10	3.31	74.41	1.15	3.33
	WWH	75.36	1.12	3.62	76.05	1.21	3.63	76.72	1.30	3.67
	Average	73.18	1.02	3.38	73.83	1.09	3.42	74.47	1.15	3.42
	QEH	75.54	1.07	3.56	76.15	1.15	3.57	76.79	1.23	3.60
	QMH	77.16	0.99	3.70	77.75	1.04	3.72	78.45	1.13	3.74
AD-Net	XJH	76.80	0.82	3.42	77.46	0.91	3.45	78.13	0.97	3.45
	WWH	77.02	1.23	3.89	77.54	1.32	3.92	78.13	1.39	3.92
	Average	76.37	1.05	3.62	76.96	1.12	3.65	77.57	1.18	3.68
	QEH	74.77	1.02	3.50	75.39	1.08	3.52	76.00	1.16	3.56
	QMH	76.03	1.13	3.33	76.69	1.21	3.33	77.37	1.30	3.37
Multi-resU-Net	XJH	77.86	1.08	3.56	78.37	1.15	3.58	78.87	1.25	3.59
	WWH	76.93	0.86	3.20	77.53	0.92	3.25	78.18	0.97	3.26
	Average	76.41	1.04	3.37	76.99	1.11	3.41	77.60	1.17	3.45
	QEH	78.09	1.06	4.46	78.62	1.15	4.46	79.13	1.25	4.48
	QMH	77.24	0.98	4.35	77.74	1.05	4.36	78.39	1.14	4.36
nnformer	XJH	74.37	1.12	7.26	74.96	1.18	7.27	75.65	1.27	7.27
	WWH	75.94	1.04	4.27	76.64	1.12	4.31	77.20	1.19	4.35
	Average	76.20	1.05	4.29	76.89	1.14	4.33	77.59	1.21	4.37
	QEH	76.92	0.87	4.06	77.49	0.97	4.07	78.13	1.02	4.09
	QMH	77.60	1.15	4.22	78.29	1.24	4.26	78.97	1.32	4.27
nnU-Net	XJH	77.17	1.02	4.30	77.85	1.10	4.34	78.51	1.17	4.35
	WWH	76.78	1.09	4.80	77.47	1.18	4.82	78.07	1.27	4.84
	Average	77.12	1.07	4.32	77.72	1.14	4.37	78.42	1.20	4.39
	QEH	79.85	1.07	3.73	81.68	1.20	4.34	82.23	1.28	4.67
	QMH	76.91	0.90	3.39	79.19	1.04	4.05	80.07	1.13	4.45
Proposed	XJH	79.60	0.83	3.67	81.78	0.94	4.36	82.78	1.04	4.72
	WWH	79.38	1.32	3.85	81.34	1.46	4.41	82.01	1.56	4.91
	Average	**78.99**	**1.02**	**3.81**	**80.97**	**1.16**	**4.34**	**81.77**	**1.25**	**4.69**

## Data Availability

The data that support the findings of this study are confidential.
